# Direct RNA Sequencing Reveals Sex-Biased Transcriptomic and Epitranscriptomic Regulation in *Procambarus clarkii*

**DOI:** 10.3390/biology14121757

**Published:** 2025-12-08

**Authors:** Haijing Xu, Guangtong Song, Yichen Luo, Haoxuan Zhang, Muhammad Jawad, Wei Zhang, Tao Li, Dawei Zhao, Chunyan Yang, Aimin Wang, Mingyou Li

**Affiliations:** 1 Key Laboratory of Exploration and Utilization of Aquatic Genetic Resources by the Ministry of Education, Shanghai Ocean University, Shanghai 201306, China; 2 Institute of Fisheries, Anhui Academy of Agriculture Science, Hefei 230001, China; 3 Jiangsu Mingzhi biotechnology Co., Ltd., Yancheng 224100, China; 4 Yandu Fishery Technology Extension Station, Yancheng 224002, China; 5 Key Laboratory of Aquaculture and Ecology of Coastal Pool of Jiangsu Province, College of Economics, Yancheng Institute of Technology, Yancheng 224007, China

**Keywords:** *Procambarus clarkii*, Direct RNA Sequencing (DRS), sex determination, epitranscriptomics, gonadal transcriptome

## Abstract

The red swamp crayfish (*Procambarus clarkii*) is a globally important freshwater crustacean that exhibits pronounced sexual dimorphism, with males growing faster than females. However, the molecular mechanisms underlying sex differentiation in crustaceans remain poorly understood. In this study, Oxford Nanopore-based Direct RNA Sequencing (DRS) was employed to analyze the gonadal transcriptomes of male and female *P. clarkii*, identifying 20,001 previously unannotated genes and revealing extensive sex-specific differences in transcript structure, alternative splicing, and RNA modifications. Ovarian transcripts had shorter poly(A) tails and more frequent alternative splicing, while male gonads showed greater enrichment of m^6^A and psU modifications in the 3′ UTRs. qPCR validation confirmed the sex-biased expression of key candidate genes, including *Dmrt7*, *FR*, *Fruitless*, *IAGBP*, *RDH*, and *Vtg*. Collectively, these findings provide the first comprehensive epitranscriptomic landscape of *P. clarkii* gonads, underscoring the pivotal role of post-transcriptional regulation in sex determination and offering valuable insights for mono-sex breeding strategies in aquaculture.

## Simple Summary

This study, utilizing Direct RNA Sequencing technology, systematically characterized the full-length transcript architecture, alternative splicing patterns, and RNA methylation profiles within the gonadal transcriptome of *P. clarkii*. This work addressed the current gaps in transcript annotation and gender-specific post-transcriptional regulatory mechanisms in this species. This study is the first to uncover sex-associated differential splicing events and epitranscriptomic regulatory disparities between males and females, providing crucial data support for a deeper understanding of the molecular basis of sex determination in *P. clarkii*. These findings establish a significant theoretical foundation and offer promising practical applications for the development of sex-control breeding technologies.

## 1. Introduction

*Procambarus clarkii*, also known as the red swamp crayfish, has become one of the most commercially important freshwater crustaceans worldwide, particularly in China. Its strong adaptability, rapid growth rate, and distinctive flavor have driven the establishment of a complete industrial chain encompassing large-scale aquaculture, processing, and a substantial catering and consumer market. By 2024, the national output value of *P. clarkii* in China exceeded 500 billion RMB, creating millions of jobs and making it a staple of the national food culture [1]. Despite this remarkable growth, the industry faces pressing challenges, among which sexual dimorphism is particularly significant. Although male individuals typically achieve larger body sizes, their higher proportion of cephalothorax and claw mass reduces the yield of edible muscle. Consequently, females often have greater market value [2]. This growth disparity directly influences aquaculture profitability and amplifies demand for female-biased breeding production.

Mono-sex farming strategies have already demonstrated substantial industrial value across aquaculture. For instance, all-male populations of *Pelteobagrus fulvidraco* and *Oreochromis niloticus* increase production by 30–50% [3,4,5,6]. In the crustacean species *Macrobrachium rosenbergii*, single-sex breeding offers dual advantages: all-male populations benefit from the greater growth potential of males, enabling the production of super-large-sized commercial prawn and significantly increasing market value [5,7], whereas all-female populations exhibit more uniform individual sizes and reduced cannibalism, resulting in a 20% to 30% increase in yield per unit [6,8]. These studies underscore that sex-specific breeding strategies, tailored to the biological characteristics of the species, are key to improved farming efficiency.

Elucidating the molecular mechanisms of sex differentiation is therefore critical for advancing mono-sex aquaculture. In crustaceans, the insulin-like androgenic gland hormone (IAG) has received the most attention. Functional disruption of IAG induces male-to-female sex reversal in *M. rosenbergii* [7,8], *Exopalaemon carinicauda* [9], and *M. nipponense* [10]. However, similar manipulations in *P. clarkii* and *Penaeus vannamei* did not alter sexual phenotype [11,12,13]. These contrasting outcomes suggest that sex regulation in *P. clarkii* involves pathways beyond or parallel to the IAG axis [11,12]. Although Crustacea and Insecta both belong to the phylum Arthropoda and share a close evolutionary relationship, research on their sex-determination mechanisms has progressed very differently. In insects such as *Drosophila melanogaster* and *Bombyx mori*, sex differentiation is governed by highly conserved post-transcriptional cascades of alternative splicing, exemplified by the regulatory roles of *Sxl* and *Tra* [14]. Homologs of these sex-regulating genes have been identified in crustaceans, but their functional involvement in sex determination remains uncertain. Importantly, recent studies indicate that post-transcriptional regulation may indeed contribute to crustacean sexual differentiation: in the branchiopod *Daphnia magna*, sex-specific alternative splicing of *Dsx1* is central to environmental sex determination [15,16], and full-length transcriptome sequencing of decapod gonads has revealed widespread isoform diversity and sex-biased splicing events. These findings imply a role for alternative splicing in crustacean sex regulation, though the precise cascades, analogous to those in insects, remain uncharacterized.

This knowledge gap is largely due to the limitations of short-read sequencing technologies, which are insufficient for accurately resolving full-length splice isoforms. With recent advances in sequencing technologies, third-generation platforms such as Oxford Nanopore Technologies (ONT) (Oxford, UK) now enable unbiased full-length transcriptome profiling. In particular, direct RNA sequencing (DRS) allows not only precise identification of transcript isoforms but also simultaneous detection of RNA modifications, thereby offering a powerful tool for investigating post-transcriptional regulatory mechanisms during specific physiological processes.

This study aims to apply DRS-based third-generation sequencing to systematically characterize sex-related differences in transcript structure, alternative splicing, and RNA modification between male and female *P. clarkii*, aiming to uncover the potential roles of post-transcriptional and epitranscriptomic mechanisms underlying sex differentiation in this species.

## 2. Materials and Methods

### 2.1. P. clarkii Sample Collection

**Figure 1:**
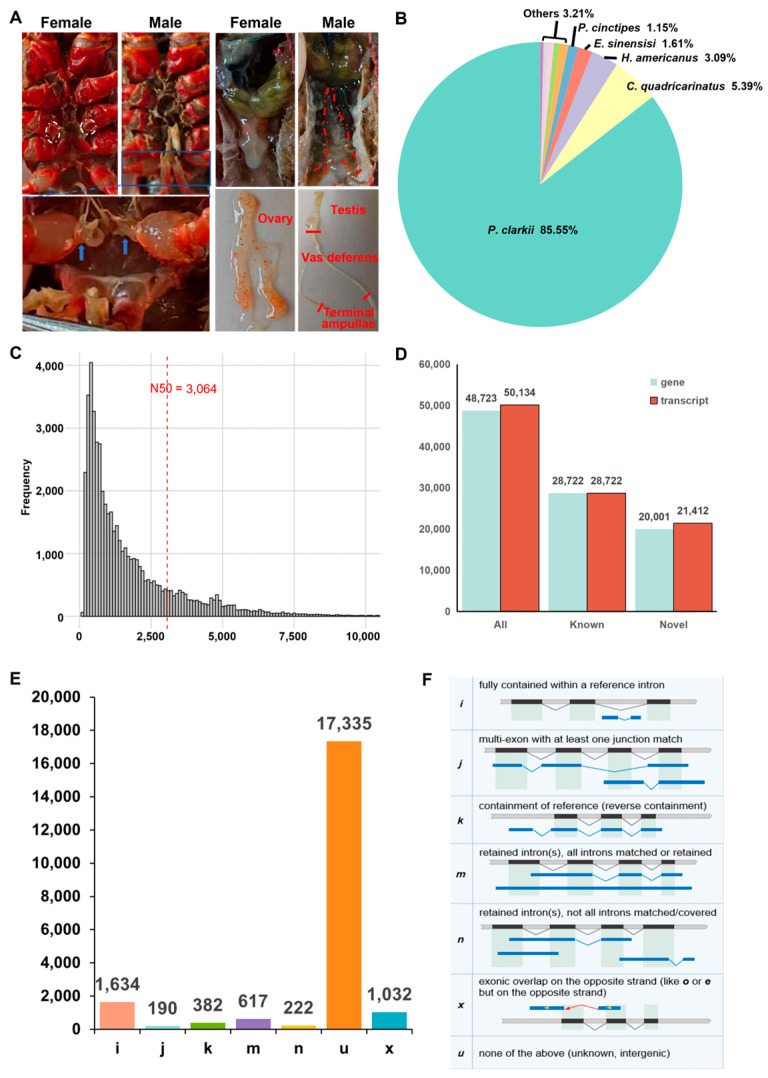
Statistics of direct RNA sequencing data of from *P. clarkii* gonads. (**A**) External sexual characteristics and anatomical structures of the male and female reproductive system. The white dotted oval marks the region of the female gonopore. The blue arrow points to the terminal ampulla. The red dotted line outlines the testis and vas deferens. (**B**) Species distribution of transcript annotations in the NR database. (**C**) Length distribution of coding sequences (CDS) in newly identified transcripts, with the red dotted line representing the N50 length. (**D**) Transcript and gene counts in the transcriptome. (**E**) The number of 7 new transcript types. (**F**) The structure of 7 new transcript types.

The crayfish used in this study belonged to the breeding strain of Yancheng Institute of Technology and were originally sourced from Yancheng City, Jiangsu Province. Prior to sampling, *P. clarkii* specimens were anesthetized on ice. Mature male individuals used in the experiment had a mean body weight of 44.0 ± 2.0 g, while mature females had a mean body weight of 28.6 ± 2.0 g; specifically, females at ovarian developmental stages III–IV were selected for DRS analysis. Subsequently, six male reproductive systems and six ovaries (Figure 1A) were dissected and pooled separately. For each sex, gonads from three individuals were pooled into one tube, generating two technical replicates for males (labeled Male reproductive system 1 and Male reproductive system 2) and two for females (labeled Ovary1 and Ovary2). Additional organ samples for qRT-PCR analysis were collected from three independent female and three independent male individuals.

### 2.2. Total RNA Extraction and mRNA Enrichment

Total RNA was extracted using the Total RNA Kit (R6834 Total RNA Kit I) (Omega, Norcross, GA, USA) following the manufacturer’s instruction. RNA concentration and purity were assessed using a NanoDrop spectrophotometer (Thermo Fisher Scientific, Waltham, MA, USA), and integrity was verified by agarose gel electrophoresis. One microgram of RNA per sample was quantified for quality control, and 20 µg of high-quality RNA was used for direct RNA sequencing. mRNA enrichment was performed using the NEBNext Poly(A) mRNA Magnetic Isolation Module (E7490S) (NEB, Ipswich, MA, USA) according to the manufacturer’s instructions.

### 2.3. Nanopore Sequencing Library Construction and Sequencing

Prepared mRNA was used for DRS library construction following the Oxford Nanopore DRS protocol (SQK-RNA004, Oxford Nanopore Technologies, Oxford, UK). For reverse transcription adapter ligation, 9 μL of prepared mRNA was mixed with 3 μL NEBNext Quick Ligation Reaction Buffer (NEB, Ipswich, MA, USA), 1 μL RT Adapter (RTA) (SQK-RNA004, Oxford Nanopore Technologies, Oxford, UK), and 2 μL T4 DNA Ligase (NEB, Ipswich, MA, USA), and then incubated at 24 °C for 10 min. Subsequently, 8 μL 5× first-strand buffer (NEB, Ipswich, MA, USA), 2 μL 10 mM dNTPs (NEB, Ipswich, MA, USA), 9 μL nuclease-free water, 4 μL 0.1 M DTT (Thermo Fisher Scientific, Waltham, MA, USA), and 2 μL SuperScript III Reverse Transcriptase (Thermo Fisher Scientific, Waltham, MA, USA) were added to the 15 μL reaction system. The mixture was incubated at 50 °C for 50 min followed by 70 °C for 10 min. Reverse-transcribed mRNA was purified with 1.8× volume of Agencourt RNAClean XP beads (Beckman Coulter Life Sciences, Indianapolis, IN, USA) and eluted in 23 μL nuclease-free water. Then, 8 μL NEBNext Quick Ligation Reaction Buffer (NEB, Ipswich, MA, USA), 6 μL RNA Adapter (RLA) (Oxford Nanopore Technologies, Oxford, UK), and 3 μL T4 DNA Ligase were added for sequencing adapter ligation (24 °C, 10 min). The product was purified as above and eluted in 32 μL RNA Elution Buffer (SQK-RNA004, Oxford Nanopore Technologies, Oxford, UK). A total of 100 μL SB (SQK-RNA004, Oxford Nanopore Technologies, Oxford, UK) and 68 μL LIS (SQK-RNA004, Oxford Nanopore Technologies, Oxford, UK) were added to the eluate. The final mixture was loaded onto a Nanopore FLO-PRO004RA flow cell and sequenced for 48–72 h using a PromethION sequencer (Oxford Nanopore Technologies, Oxford, UK). This experiment was completed by Wuhan Benagen Technology Co. (Benagen, Wuhan, China).

### 2.4. Preprocessing, Alignment, and Novel Gene/Transcript Analysis

Raw reads were basecalled using Dorado (version: latest; parameters: --estimate-poly-a) to assess read quality. Low-quality reads (Q < 10) were filtered during basecalling. Clean reads from each library were aligned to the *P. clarkii* genome using Minimap2 (v2.17-r941), with GenBank assembly number: GCA_040958095.1 (www.ncbi.nlm.nih.gov/datasets/genome/GCF_040958095.1/, accessed on 31 July 2024). Alignment rates to reference genes were calculated using Samtools (v1.10). Consensus sequences were obtained from alignment results using Flair (v1.5.0; parameters: -t 20). Non-redundant transcript sequences were assembled using StringTie (v2.1.4; parameters: --conservative -L -R). Transcripts were compared to known genomic transcripts using gffcompare (v0.12.1; parameters: -R -C -K -M) to identify novel transcripts and genes. For comprehensive functional annotation of novel transcripts, sequences were annotated against 7 databases (NR, Pfam, UniProt, KEGG, GO, KOG/COG, PATHWAY) based on sequence and motif similarity.

### 2.5. Transcript Structure Analysis

Transcript boundaries were corrected by extending untranslated regions (UTRs) beyond annotated regions where supported by sequencing evidence. Alternative splicing (AS) events for each sample were identified using Suppa2 (v2.3; parameters: --boundary S -f ioe -e SE SS MX RI FL), with differential AS between groups detected via Suppa2 (DiffSplice). Fusion transcripts were identified using FusionSeeker (v1.0.1). Coding potential of newly identified transcripts was predicted using CNCI (v2.0; default parameters), CPC2 (standalone_python3 v1.0.1), and PLEK (v1.2).

### 2.6. Isoform Poly(A) Length Analysis

Aligning reads were processed to extract alignment end positions from BAM files. Poly(A) sites were identified, clustered, and annotated using Quantifypoly(A). Differences in poly(A) tail lengths were calculated from valid data using Dorado (v1.3.0; parameters: --estimate-poly-a). Mann–Whitney U tests were performed to assess poly(A) length differences at both global and transcriptome levels between groups. |Diff_Median| (difference in median poly(A) lengths) indicated effect size/direction, and FDR (false discovery rate) determined significance. Transcripts with FDR < 0.05 and |Diff_Median| > 15 were considered differentially polyadenylated.

### 2.7. RNA Methylation Analysis

Modified sites (m^6^A/psu/m5c/inosine) were detected using Dorado’s latest model (rna004_130bps_sup@v5.1.0). Differential methylation loci (DML) and transcript analyses were performed using modkit (v0.4.1). Differential methylation was tested by logistic regression, applicable even for single-sample groups.

The subsequent quantification of cDNA products was performed as described previously [17]. The primers for target gene qRT-PCR are provided in Supplementary Table S1.

### 2.8. Statistical Analysis

All the experimental data from at least three independent experiments were analyzed using GraphPad Prism 7.0 software (San Diego, CA, USA) and were presented as mean ± SD.

## 3. Results

### 3.1. Statistics of DRS Data of the Gonads of P. clarkii

To assess the sequencing quality of DRS, the main sequencing indicators for male and female gonadal samples of *Procambarus clarkii*, including sequencing data volume (Seq Num), average length (Mean length, bp), N50 (bp), maximum transcript length (Max length, bp), and mapping rate (Map rate), are summarized in Table S2. Across all samples, the effective sequencing yield per group was approximately 6.5 GB (Table 1). On average, male gonadal samples yielded approximately 10 million reads per replicate, whereas female ovarian samples generated approximately 6.5 million reads per replicate (Table 1). The number of transcripts detected in the male reproductive system was 28,225 and 29,386, respectively, significantly higher than the 22,276 and 23,583 detected in the female ovary. In contrast, the average length of transcripts in the female ovary was approximately 1350 bp, longer than the approximately 1200 bp in the male gonad; the longest transcript lengths detected in the ovary were 13,785 and 13,785 Bp, respectively, also significantly longer than the approximately 10,000 bp in the male reproductive system. The overall quality of this DRS was high, with an N50 value of approximately 2500 bp, and the genome mapping rate of all samples exceeded 90%.

Annotation analysis of the transcripts obtained from this sequencing in the Nr database revealed that the top five species with the highest sequence similarity are all crustaceans, collectively accounting for 96.79% of all annotated data. Notably, 85.55% of the transcripts were specifically annotated to *P. clarkii* (Figure 1B). A total of 48,723 genes were identified, corresponding to 50,134 transcript sequences. Among these, 28,722 genes were previously annotated, each producing a single transcript, whereas the remaining 20,001 genes generated 21,412 transcripts, potentially representing novel genes or genes with multiple isoforms. To further classify the newly identified transcripts, gffcompare software was used to compare the assembled transcripts with the reference genome annotations, thereby refining and expanding the transcriptomic annotation. This analysis identified seven novel transcript types (I, J, K, M, N, U, and X), totaling 201,412 transcripts. Among them, U-type transcripts were most abundant, with 173,350 entries, highlighting the substantial incompleteness of the current *P. clarkii* transcript annotation and the need for further refinement.

### 3.2. Differential Gene Expression Analysis Between Gonads

**Figure 2:**
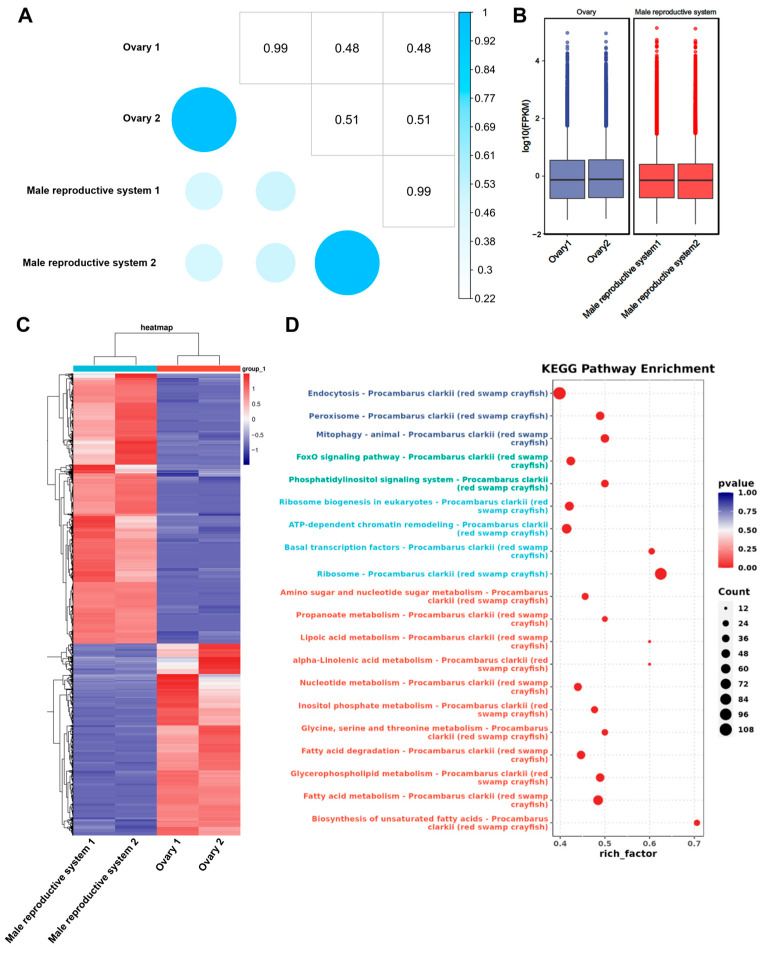
Comparative analysis of DRS transcript profiles in female and male gonads. (**A**) Correlation analysis on the different samples. (**B**) Comparative analysis of global transcriptomic expression levels in male and female gonads of *P. clarkii*. (**C**) Heatmap representation of clustering analysis for differentially expressed transcripts. (**D**) KEGG enrichment analysis of differentially expressed transcripts in male and female gonads.

Although only two technical replicates were set up for this DRS, replicate consistency was high, with correlation coefficients exceeding 0.99 for both ovarian and testicular samples. The overall transcriptional intensity was markedly higher in the ovary compared with the testis (Figure 2B), likely reflecting the accumulation of maternal transcripts required to support early embryonic development.

Compared with the ovary, 8003 genes were significantly up-regulated and 11,299 genes were significantly down-regulated in the male reproductive system (Figure 2C). KEGG pathway enrichment analysis of differentially expressed genes showed that the top 20 significantly enriched signaling pathways included endocytosis, FoxO signaling pathway, fatty acid metabolism, and others. These pathways exhibited pronounced sex-specific expression patterns and are likely involved in the regulation of gonadal physiology and maintaining sex-specific functions.

### 3.3. Structure Analysis of P. clarkii Gonads

**Figure 3:**
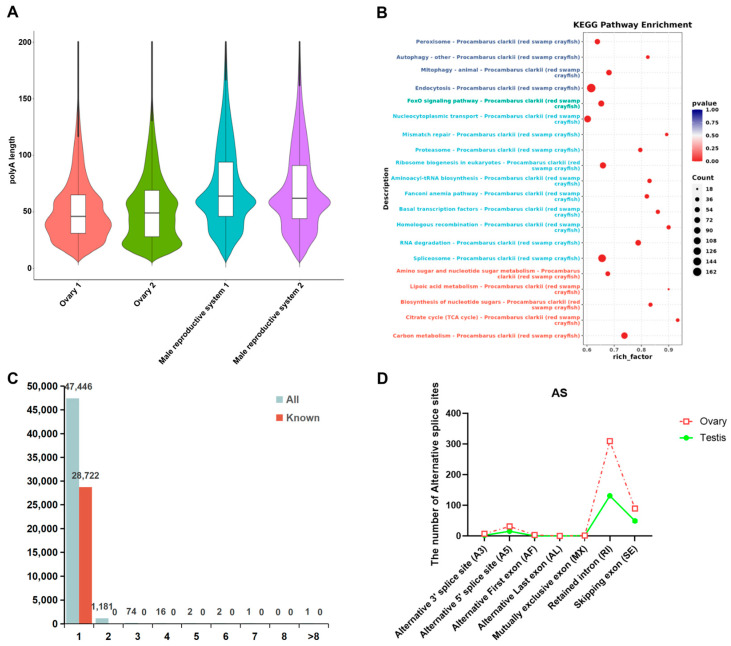
Comparative analysis of transcriptomic structural variations in male and female gonads of *P. clarkii*. (**A**) Distribution of Poly(A) tail lengths in male and female gonads of *P. clarkii*. (**B**) KEGG enrichment analysis of differential transcripts with different Poly(A) tail lengths in male and female gonads. (**C**) Statistical analysis of the number of transcripts contained in different genes. (**D**) The number of alternative splice sites of male and female gonads.

Transcripts from female and male gonads exhibited significant differences in poly(A) tail length, with ovarian transcripts displaying substantially shorter poly(A) tails compared to those from the male reproductive system (Figure 3A). This pattern suggests that maternal mRNA stored in the oocytes possess shorter poly(A) tails, rather than the extended tails, poly(A) tail structures, whereas transcripts in the male reproductive system retain poly(A) tails of typical length (Figure 3A). To investigate the functional implications of these differences, KEGG pathway enrichment analysis was conducted on transcripts with variable poly(A) tail lengths. The results revealed significant enrichment in pathways associated with cellular processes, genetic material processing, and metabolic regulation. Notably, pathways related to genetic material metabolism showed the highest number of enriched terms, which aligns closely with the biological process of maternal mRNA accumulation and storage in developing oocytes (Figure 3B).

In the DRS analysis of male and female *P. clarkii*, a total of 1277 genes displayed distinct splicing patterns, which fell into two main categories: 1181 genes with two distinct splicing isoforms and 74 genes involved in alternative splicing events (Figure 3C). Among the differentially expressed transcripts in male and female gonads, 309 genes were identified as undergoing differential splicing, encompassing 675 transcript variants (Table S3). Furthermore, we analyzed the types of alternative splicing present in each sample and quantified the corresponding transcript counts. As illustrated in Figure 3D, significant differences were observed in the distribution of specific alternative splicing events—such as alternative 5’ splice sites (A5), intron retention (RI), and exon skipping (SE)—between the ovaries and the male reproductive system. Notably, the ovaries exhibited a markedly higher number of alternative splicing events compared to the male reproductive system.

### 3.4. RNA Modification Analysis of P. clarkii Gonads

**Figure 4:**
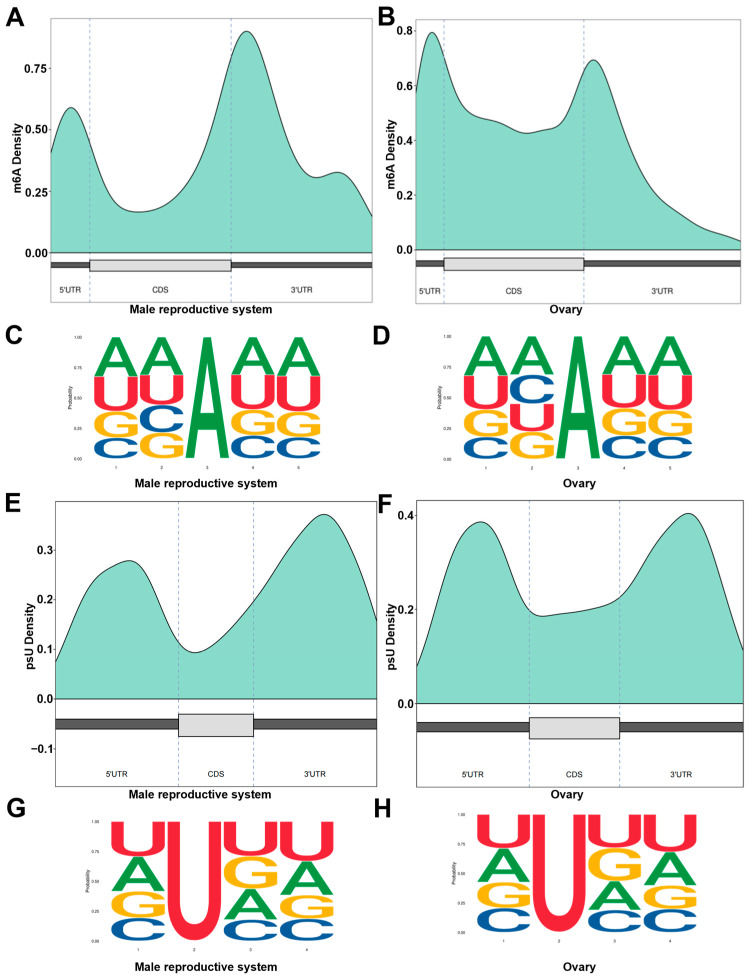
Methylation analysis of male and female gonadal transcripts of *P. clarkii.* (**A**,**B**) The percentage of m^6^A methylation annotations at different positions. (**C**,**D**) The genetic features of the 5 bp long m^6^A methylation sites in male and female gonads. (**E**,**F**) The percentage of psU modification annotations at different positions. (**G**,**H**) The genetic features of the 5 bp long psU modification sites in male and female gonads.

Based on the reference genome, functional annotations of m^6^A and psU modifications were performed within gene coding regions. The distribution of m^6^A sites (i.e., the methylation annotation ratio) showed that in the male reproductive system, modification sites were predominantly enriched in the 3′ UTR (Figure 4A), whereas in the ovary, a considerable proportion were also present (Figure 4B). Similarly, the proportional analysis of psU modifications sites in males was mainly concentrated in the 3′ UTR (Figure 4C), while in the ovary, the psU modifications were more evenly distributed across the 5′ UTR and 3′ UTR (Figure 4D). Additionally, the analysis of the base sequence characteristics before and after m^6^A and psU modifications revealed that the second base of the m^6^A methylation sites exhibited different sequence preferences in the male reproductive system and ovarian tissues, while the base sequence characteristics upstream and downstream of the psU modification sites remained consistent in both tissues without significant changes.

### 3.5. Verification of Potential Sex-Related Genes

**Figure 5:**
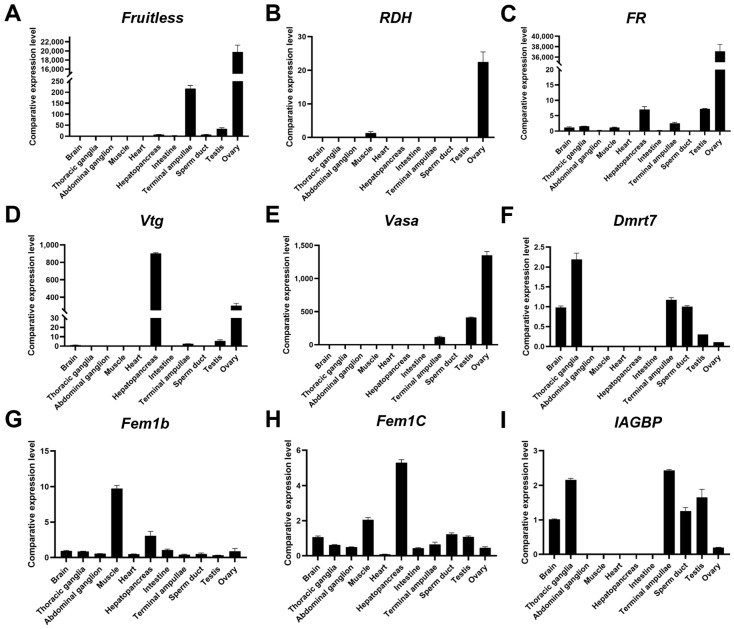
qPCR verification of sex-related genes. (**A**–**D**) *Fruitless*, *RDH*, *FR*, and *Vtg* are termed female-biased genes. (**E**) *Vasa* is termed a gonad-characterizing gene. (**F**,**I**) *Dmrt7* and *IAGBP* are termed male-biased genes. (**G**,**H**) *Fem1b* and *Fem1C*, classical female-associated genes, showed no significant differential expression between sexes in transcriptomic and qPCR assays.

To identify potential genes associated with gonadal differentiation and development in *P. clarkii*, we selected *Fruitless*, *retinol dehydrogenase* (*RDH*), and *folate receptor* (*FR*) as candidate genes related to female differentiation, and *vitellogenin* (*Vtg*) as a key gene involved in ovarian development(Figure 5A–D). These selections were based on transcriptomic analysis of sex-biased expression and supported by previous studies in crustaceans. All of these genes exhibited ovarian-preferential expression in the transcriptomic data. For male-related genes, *Dmrt7* (the homolog of the *iDMY* gene in *Sagmariasus verreauxi*) and *IAGBP* were identified as potential regulator of male differentiation-related genes(Figure 5F,I). Although these genes are not exclusively expressed in the reproductive system, qPCR validation confirmed their preferential expression in the male reproductive system. In contrast, *Fem1b* and *Fem1c* showed no significant differential expression between sexes in either the transcriptome or qPCR assays(Figure 5G,H), indicating that they are unlikely to play roles in sex determination or differentiation in *P. clarkii*.

## 4. Discussion

*Procambarus clarkii*, as a globally important freshwater economic species, has experienced rapid industrial growth in China and worldwide, forming a complete production chain covering breeding, processing and consumption, with an annual output value of hundreds of billions of yuan [1]. This development has promoted regional economies and generated substantial employment. However, the sustainable growth of this industry is still constrained by a lack of fundamental biological knowledge, particularly regarding its pronounced sexual dimorphism. While the traditional view emphasizes the larger body size and faster growth of males, recent studies demonstrate that females offer unique advantages including higher meat yield, greater body-size uniformity, and better suitability for processing traits that better align with the requirements of intensive aquaculture and standardized production [18]. Despite this, the molecular mechanism of sex differentiation in crustaceans remain poorly understood.

Currently, sex control strategies in decapods have primarily focused on the insulin-like androgenic gland hormone (*IAG*). Functional disruption of *IAG* has successfully induced male-to-female sex reversal in *Macrobrachium rosenbergii* and other palaemonid prawns [7,9,10]. However, similar approaches have not been successful in *P. clarkii* or *P. vannamei* [11,12,13], underscoring the diversity of sex-determination mechanisms across crustaceans. Although a comparative study on the gonadal transcriptome of *P. clarkii* was conducted in 2014 [19], progress was limited by the sequencing technologies available at the time and the absence of a reference genome, which only became available in 2021 [20,21]. In this study, Direct RNA Sequencing (DRS) technology was employed to systematically analyze transcript composition, alternative splicing patterns and RNA methylation modifications in male and female gonads of *P. clarkii*. This work not only identified 20,001 novel genes, substantially expanding transcriptome annotation, but also revealed sex-specific splicing events and differences in RNA modifications, providing the first evidence for epitranscriptomic regulation in crayfish gonads. These findings provide a key data foundation for in-depth analysis of its sex determination mechanism and have significant theoretical value and application prospects for promoting the development of sex control breeding technology in *P. clarkii*.

Mechanisms of sex determination in invertebrates are highly diverse and differ markedly from vertebrates. In vertebrates, the differentiation of female and male gonads is primarily governed by the antagonistic interaction between estrogen and androgen [22]. In contrast, no regulatory system analogous to the sex steroid hormone-dominated system in vertebrates has been identified in invertebrates, and their sex determination mechanisms exhibit considerable complexity and diversity. For example, in Diptera species such as *D. melanogaster*, sexual differentiation is initiated by the alternative splicing of the *sxl* gene [14,23]. In contrast, in Lepidoptera species like *Bombyx mori*, which are also arthropods, sex determination is predominantly regulated by the interaction between the *Masc* gene and *fem* piRNA [24]. Overall, in most species within the class Insecta, alternative splicing of sex-related genes plays a crucial role in sex determination [25]. In Daphnia magna, sex determination involves sex-specific splicing of *Dsx1* isoforms, providing direct evidence of splicing-mediated sex regulation in crustaceans [15]. Our DRS results revealed extensive isoform diversity and sex-biased splicing events, particularly in ovaries (Table S3; Figure 3), reinforcing the hypothesis that alternative splicing contributes to gonadal differentiation in *P. clarkii*. Notably, among the differentially expressed transcripts in male and female gonads, 309 genes were identified as undergoing differential splicing, encompassing 675 transcript variants. Among the differentially expressed genes, 675 transcripts derived from 309 genes exhibited differential expression in female and male gonads, with 75 splicing isoforms encoded by 36 genes showing opposing expression patterns between the sexes (Table S3).

Epigenetic regulation and genetic programs act in concert to determine the differentiation trajectory of gonads in both vertebrates and invertebrates [22,26]. RNA methylation, such as m^6^A, serves as a critical post-transcriptional epigenetic regulatory mechanism and has been extensively demonstrated to precisely modulate RNA alternative splicing [27,28], stability [29], and translation efficiency [30]. Studies have revealed that m^6^A modification in female neurons of *D. melanogaster* determines sexual fate by regulating the alternative splicing of the *Sxl* gene [14,23]. This function relies on the coordinated activity of methyltransferase complexes (*Mettl3*) and reader proteins (*YTHDC1*), and can be finely tuned by factors such as *Nab2* through the inhibition of m^6^A deposition [31,32]. In this study, using Direct RNA Sequencing (DRS), we identified a set of candidate genes associated with sex differentiation that exhibit sexually dimorphic m^6^A methylation (Table S5) and psU modification patterns (Table S6). These findings provide novel insights into the epitranscriptomic regulatory mechanisms underlying sex determination in *P. clarkii*. Poly(A) tail length is another post-transcriptional regulatory layer with important developmental implications. In our study, ovarian transcripts displayed significantly shorter poly(A) tails compared to those from testis, consistent with the role of polyadenylation in maternal mRNA storage during oocyte maturation and early embryogenesis [33]. This observation suggests that post-transcriptional control of transcript stability and translation timing is a key feature of ovarian physiology in *P. clarkii*.

To verify candidate sex-related genes, qPCR analysis confirmed that *Fruitless*, *RDH*, *FR*, and *Vtg* exhibited ovarian-preferential expression, consistent with transcriptome data and supporting their roles in female gonadal development. Conversely, *Dmrt7* (a homolog of *iDMY* in *Cherax quadricarinatus*) [34]) and *IAGBP* displayed male-biased expression, suggesting roles in testis differentiation. However, in agreement with the transcriptome data, *Fem1b* and *Fem1c* did not exhibit significant sex-biased expression in the qPCR analysis, suggesting that their involvement in sex determination in crayfish may follow a mechanism distinct from the fem gene-mediated sex regulation pathway observed in insects. These experimentally validated genes represent high-priority candidates for future functional studies, including gene knockdown or overexpression experiments.

## 5. Conclusions

This study employed Oxford Nanopore Direct RNA Sequencing to systematically characterize the sex-biased transcriptomic and epitranscriptomic landscapes in the gonads of the red swamp crayfish, *Procambarus clarkii*. Significant differences were observed between males and females: ovarian transcripts exhibited shorter polyA tails and more frequent alternative splicing events, whereas testicular transcripts showed distinct enrichment of m^6^A and psU modifications in their 3′ UTRs. qPCR validation confirmed the sex-biased expression of key candidate genes involved in gonadal differentiation. These findings present the first comprehensive epitranscriptomic profile of *P. clarkii*, highlighting the critical role of post-transcriptional regulation in sex determination and providing a robust molecular foundation for the development of mono-sex breeding strategies in crustacean aquaculture.

## Figures and Tables

**Table 1:** Statistics of DRS Data.

Sample	Type	Total Base	Total Reads	Max Len.	Avg. Len.	N50	L50	N90	L90
Ovary 1	all	6.71 Gb	6.86 Mb	352,878	1001.8	1333	1.51 Mb	587	4.47 Mb
Ovary 1	pass	6.55 Gb	6.28 Mb	352,878	1067.61	1337	1.48 Mb	600	4.34 Mb
Ovary 2	all	6.39 Gb	6.80 Mb	447,796	961.94	1268	1.54 Mb	568	4.48 Mb
Ovary 2	pass	6.22 Gb	6.18 Mb	447,796	1031.09	1270	1.50 Mb	581	4.30 Mb
Male reproductive system 1	all	6.51 Gb	11.13 Mb	426,539	598.9	1007	2.01 Mb	332	6.41 Mb
Male reproductive system 1	pass	6.26 Gb	9.5 Mb	426,539	673.61	1010	1.96 Mb	347	6.11 Mb
Male reproductive system 2	all	6.79 Gb	12.17 Mb	429,741	571.24	962	2.24 Mb	318	7.04 Mb
Male reproductive system 2	pass	6.57 Gb	10.18 Mb	429,741	661.1	971	2.17 Mb	333	6.71 Mb

## Data Availability

Data contained within the article. The raw data of this work was uploaded to NGDC (https://ngdc.cncb.ac.cn) with BioProject number PRJCA049565, and will become available from 28 October 2026.

## Supplementary Materials

The following supporting information can be downloaded at: https://www.mdpi.com/article/10.3390/biology14121757/s1. Table S1: Primers used for RT-qPCR. Table S2: The number of sequencing reads of different samples. Table S3: Differentially expressed isoforms in gonads. Table S4: Key genes related to sex differentiation. Table S5: Genes with sexually dimorphic m6A methylation. Table S6: Genes with sexually dimorphic psU modification.
