# Supplementary material for: Direct RNA Sequencing Reveals Sex-Biased Transcriptomic and Epitranscriptomic Regulation in Procambarus clarkii
Source: Biology (Basel). 2025 Dec 8;14(12):1757. doi: 10.3390/biology14121757 (PMC12731033; doi:10.3390/biology14121757)
Supplement: Supplementary file 1 [file biology-14-01757-s001.zip › +Table S1 primers.pdf]

Table S1 Primers used for RT-qPCR

Primer name	sequence	Product size	Accession No.
Pc-actin-qF	5’- CAGGGCGTGATGGTTGGT-3’	200 bp	KR135165.1
Pc-actin-qR	5’- TCCGTCAGCAGGACTGGGTG-3’		
Pc-FR-qF	5’-AGTGTCACCAGTGGCAGAATAG	233 bp	XM_045739853.2
Pc-FR-qR	5’-CAGAGGCACGTTCCTGTATC-3’		
Pc-RDH-qF	5’-AGAAACGCACCACAGCAGATG-3’	185 bp	XM_045737355.2
Pc-RDH-qR	5’-CACGTCGTAGTTAAGGTCATCCG-3’		
Pc-Fru-qF	5’-GTCTCTTCGCGTCTGGAGTAAC-3’	192 bp	XM_045756879.1
Pc-Fru-qR	5’-ACTGGAAAGCAACTGGGTCG-3’		
Pc-Vtg-qF	5’-CAGAATCCAACGACCAATCAG-3’	188 bp	OK142726.1
Pc-Vtg-qR	5’-TACCAGAGCAGCAACATCACG-3’		
Pc-Vasa-qF	5’-GATCGTATGTTGGACCTGGG-3’	260 bp	XM_045759475.2
Pc-Vasa-qR	5’-GGTGATGTCTTAATCAGCGTGTC-3’		
Pc-Dmrt7-qF	5’-AACGGCGGTAGAGGTATTGTC-3’	209 bp	XM_045767807.1
Pc-Dmrt7-qR	5’-CTATGAGTGCTGTTGGGATGG-3’		
Pc-IGFBP-qF	5’-GGAGTACCTGGTTTGTCGC-3’	213 bp	XM_045767807.1
Pc-IGFBP-qR	5’-TCTTCTGGCTTGGATTTGAT-3’		
Pc-Fem1c-qF	5’-ACCTGACCAGTAGATACCACGATG-3’	221 bp	XM_045749425.2
Pc-Fem1c-qR	5’-AACTCCAGATGCCTGACGAATG-3’		
Pc-Fem1b-qF	5’-ATCTCCCTCTACGCCCACTTG-3’	188bp	XM_045764681.2
Pc-Fem1b-qR	5’-AACTTAACGGTTCCCTCTTGTTC-3’		
