# Supplementary material for: Direct RNA Sequencing Reveals Sex-Biased Transcriptomic and Epitranscriptomic Regulation in Procambarus clarkii
Source: Biology (Basel). 2025 Dec 8;14(12):1757. doi: 10.3390/biology14121757 (PMC12731033; doi:10.3390/biology14121757)
Supplement: Supplementary file 1 [file biology-14-01757-s001.zip › +Table S2.pdf]

Table S2 The number of sequencing reads of different samples.

Sample name	Seq Num	Mean length(bp)	N50(bp)	Max length(bp)	Map rate
Ovary 1	23,583	1,376.00	2,743	13,785	96.06%
Ovary 2	22,276	1,399.00	2,717	16,065	96.51%
Male reproductive system 1	28,285	1,258.00	2,486	10,633	90.56%
Male reproductive system 2	29,386	1,139.00	2,362	10,364	92.94%
