# Supplementary material for: Direct RNA Sequencing Reveals Sex-Biased Transcriptomic and Epitranscriptomic Regulation in Procambarus clarkii
Source: Biology (Basel). 2025 Dec 8;14(12):1757. doi: 10.3390/biology14121757 (PMC12731033; doi:10.3390/biology14121757)
Supplement: Supplementary file 1 [file biology-14-01757-s001.zip › Table S4 key genes related sex differentiation.pdf]

Gene Description	NR ID	Gene ID	Ovary	Testis	log2Foldchange	P value	P adj	
Uncharacterized protein (*Fru*)	XP_045612835.1	Pclarkia004059.1	2270.9410	1.1576	10.9291	1.82E-36	1.30E-34	Up
Vitellogenin 1	UFQ72463.1	Pclarkia008429.t1	2360.2205	1.1733	10.9834	7.63E-34	4.81E-32	Up
Retinol dehydrogenase 11-like	XP_045594453.1	Pclarkia003913.1	4639.3845	3.5198	10.3734	8.20E-84	3.84E-81	Up
Folate receptor alpha-like	XP_045595809.1	Pclarkia024560.1	8235.1847	24.9409	8.3700	3.14E-180	4.26E-176	Up
fem-1 homolog C-like	XP_045605381.1	novel7142.t1	58.1021	0.7926	6.2207	1.77E-07	7.83E-07	Up
fem-1 homolog B-like	XP_045620637.1	Pclarkia002671.1	264.7175	45.7315	2.5320	2.20E-14	2.82E-13	Up
Aggrecan core protein-like	XP_045610910.1	Pclarkia008207.t1	7517.6971	8.6403	9.7754	3.58E-105	2.95E-102	Up
spidroin-1-like	XP_045610918.1	Pclarkia008209.1	5928.8137	3.9004	10.5755	1.56E-93	9.44E-91	Up
Translation initiation factor IF-2	XP_045582758.1	Pclarkia026332.1	3234.8689	36.3553	6.4781	1.58E-70	4.58E-68	Up
Retinol dehydrogenase 11-like	XP_045593311.1	Pclarkia003914.1	4692.3904	1.9658	11.2373	4.28E-57	6.73E-55	Up
insulin-like growth factor 2 mRNA-binding protein 1	XP_045623763.1	XP_045619748.1	41.4669	686.9563	-4.0445	9.68E-36	6.73E-34	Down
Dmrt2	XP_045623763.1	Pclarkia022359.1	14.2395	37.6905	-1.3996	0.029	0.039	Down
Vasa	XP_045615431.1	Pclarkia028490.1	804.1180	2528.5434	-1.6531	1.55E-12	1.55E-11	Down
Dentin sialophosphoprotein-like	XP_045606533.1	novel11815.t1	2.3809	4659.1329	-10.8690	2.10E-46	2.21E-44	Down
Annexin B9-like	XP_045588033.1	Pclarkia007068.1	19.4746	7095.5773	-8.4906	1.47E-81	6.26E-79	Down
